# Gender-Based Differences in Anxiety and Depression Following Acute Myocardial Infarction

**DOI:** 10.5935/abc.20180161

**Published:** 2018-11

**Authors:** Pranas Serpytis, Petras Navickas, Laura Lukaviciute, Alvydas Navickas, Ramunas Aranauskas, Rokas Serpytis, Ausra Deksnyte, Sigita Glaveckaite, Zaneta Petrulioniene, Robertas Samalavicius

**Affiliations:** 1 Vilnius University - Faculty of Medicine, Vilnius - Lithuania; 2 Vilnius University Hospital Santaros Clinics, Vilnius - Lithuania; 3 Clinic of Emergency Medicine - Vilnius University, Vilnius - Lithuania

**Keywords:** Cardiovascular Diseases, Myocardial Infarction, Anxiety, Depression, Risk Factors, Gender Identify

## Abstract

**Background:**

Among patients with heart disease, depression and anxiety disorders are highly prevalent and persistent. Both depression and anxiety play a significant role in cardiovascular disease progression and are acknowledged to be independent risk factors. However, there is very little gender-related analysis concerning cardiovascular diseases and emotional disorders.

**Objective:**

We aimed to evaluate depression and anxiety levels in patients suffering from myocardial infarction [MI] within the first month after the MI and to assess the association between cardiovascular disease risk factors, demographic indicators and emotional disorders, as well as to determine whether there are gender-based differences or similarities.

**Methods:**

This survey included demographic questions, clinical characteristics, questions about cardiovascular disease risk factors and the use of the Hospital Anxiety and Depression Scale [HADS]. All statistical tests were two-sided, and p values < 0.05 were considered statistically significant.

**Results:**

It was determined that 71.4% of female and 60.4% of male patients had concomitant anxiety and/or depression symptomatology (p = 0.006). Using men as the reference point, women had an elevated risk of having some type of psychiatric disorder (odds ratio, 2.86, p = 0.007). The HADS-D score was notably higher in women (8.66 ± 3.717) than men (6.87 ± 4.531, p = 0.004). It was determined that male patients who developed depression were on average younger than those without depression (p = 0.005).

**Conclusions:**

Women demonstrated an elevated risk of having anxiety and/or depression disorder compared to men. Furthermore, depression severity increased with age in men, while anxiety severity decreased. In contrast, depression and anxiety severity was similar for women of all ages after the MI. A higher depression score was associated with diabetes and physical inactivity, whereas a higher anxiety score was associated with smoking in men. Hypercholesterolemia was associated with both higher anxiety and depression scores, and a higher depression score was associated with physical inactivity in women.

## Introduction

By 2020, depression is predicted to be the second highest cause of disability and mortality worldwide, surpassed only by ischemic heart disease (WHO). Myocardial infarction [MI] is a severe life-threatening event that is accompanied by an increased risk of depression and anxiety.^1^^,^^2^ A recent meta-analysis that explored the effect of the interactions of risk factors on all-cause mortality in patients with MI concluded that women have worse coronary artery disease [CAD] outcomes compared to men, with more women (17%) than men (12%) dying within 3 years of having their first MI.^3^ In addition, hospital mortality rates after acute MI have also been shown to be higher in women (16%) than in men (11%).^4^ Gender differences are correspondingly evident regarding mental stress-induced MI when assessing laboratory-based proxies, with a higher prevalence being observed in women than in men,^5^ even more so in women aged 50 years or younger.^6^ A large-scale case-control study indicated that post-MI depressive symptoms were associated with an increased risk of mortality, whereas anxiety symptoms were not an independent prognostic risk factor for new cardiovascular events or death.^7^ In contrast, another study of 5,750 patients with MI demonstrated that patients with anxiety are at a higher risk of both adverse cardiac events and all-cause mortality.^8^ The suicide risk is at its highest during the first month following discharge for MI for both patients with no history of psychiatric illness (adjusted rate ratio - 3.25) and for those with a history of psychiatric disorders (adjusted rate ratio - 64.05), with the rate ratios being comparable with those with no history of MI or psychiatric illness.^7^ The suicide risk remained higher for at least five years after the MI.^7^ Although post-MI depression is a common and burdensome condition, it remains underrecognized and undertreated.^9^^,^^10^ There are also very few gender-related analyses concerning cardiovascular diseases and emotional disorders.^11^ We, therefore, aimed to evaluate depression and anxiety levels in patients suffering from MI and to assess the association between cardiovascular disease risk factors, demographic indicators and emotional disorders, as well as to determine whether there are gender-based differences or similarities.

## Methods

### Participants and Recruitment

Patients with a documented MI who were admitted to a tertiary health care institution from 1 November 2012 to 31 May 2013 were included.

**Patients were included in the study according to the following inclusion criteria:**

1. Possessing a full understanding of the survey instructions;
2. Age > 18 years;
3. Either gender;
4. A diagnosis of acute MI verified based on two of the three standard criteria: typical chest pain, ECG presentation, elevated cardiac biomarkers;
5. Time after MI < 31 days;
6. Knowledge of Lithuanian language;
7. Completion of the survey.

**Exclusion criteria were:**

1. Cognitive impairment or physical inability to complete the survey;
2. Diagnosed depression or anxiety disorder prior to MI;
3. Antidepressant or benzodiazepine use prior to MI;
4. Patient refusal;
5. Participation in another research study.

Of the 180 patients recruited, a total of 160 patients met the inclusion criteria and were assessed. This survey included demographic questions (gender, age), clinical characteristics and questions about cardiovascular disease risk factors: diabetes mellitus, arterial hypertension, hypercholesterolemia, smoking, hypodynamia, and obesity. Furthermore, the Hospital Anxiety and Depression Scale [HADS] was used to determine anxiety and depression symptomatology. The scale contains 14 items: seven to assess anxiety and seven to assess depression. The score can be interpreted according to the following range: 0-7 - no depression or anxiety disorder; 8-10 - mild depression or anxiety disorder; 11-14 - moderate disorder; and 15-21 - severe disorder. The anxiety subscale (HADS-A) specificity is 0.78 and the sensitivity is 0.9, while the depression subscale (HADS-D) specificity is 0.79 and the sensitivity is 0.83.^12^

### Statistical analysis

The analysis was conducted using SPSS (IBM Corp. Released 2011. IBM SPSS Statistics for Windows. Version 20.0. Armonk, NY: IBM Corp) software. The Shapiro-Wilk’s test of normality was performed to verify the assumption of normality. Categorical variables were compared using the χ^2^ test. Binary logistic regression analysis and the χ^2^ test were used for categorical variables to assess the odds ratio [OR] for depression and anxiety presence associated with gender. The independent sample *t-*test, when the distribution of variables was normal, and the Mann-Whitney-Wilcoxon test, when variables showed an abnormal distribution, were used to assess continuous variables. Normally-distributed continuous variables are expressed as mean (mean ± standard deviation), whereas those with an abnormal distribution are expressed as median and interquartile range (IQR, Q1 - Q3). Correlation was assessed using Spearman’s rank correlation coefficient (p). All statistical tests were two-sided, and p values < 0.05 were considered statistically significant.

## Results

Of the 180 patients recruited, a total of 160 met the inclusion criteria (88.8%) and were assessed. A total of 101 patients (63.1%) were males and 59 (36.9%) were females. The mean age of female patients was 69.9 years, whilst the mean age of male patients was significantly lower, at 62.3 years (p < 0.001). The youngest female patient was 33 and the oldest was 92 years of age. Similarly, the youngest male patient was 26 and the oldest was 85 years of age. The overall age range of 59 years was identical for both genders. Based on the accumulated data, it was determined that 71.4% of female and 60.4% of male respondents (68.1% of all respondents) had concomitant anxiety and/or depression symptomatology (Table 1). Logistic regressions were used to assess the differences regarding the risk of each psychiatric disorder according to gender. Using men as the reference point, women had an increased risk of having some type of psychiatric disorder (odds ratio, 2.86, p = 0.007) (Table 1).

**Table 1:** Emotional disorder presentation in both genders

Emotional disorder	Men		Women		χ^2^ or β	p value
**Any emotional disorder**						
Prevalence	n = 61	60.4%	n = 48	81.4%	χ^2^ = 7.54	0.006*
Odds ratio	1		2.861 (1.33 – 6.16)		β = 1.05	0.007*
**Anxiety disorder**						
Prevalence	n = 40	39.6%	n = 38	64.4%	χ^2^ = 9.17	0.002*
Odds ratio	1		2.760 (1.42 – 5.37)		β = 1.02	0.003*
**Depression disorder**						
Prevalence	n = 48	47.5%	n = 32	54.2%	χ^2^ = 0.67	0.413
Odds ratio	1		0.764 (0.4 – 1.46)		β = 0.269	0.413
**Both depression and anxiety disorders**						
Prevalence	n = 27	26.7%	n = 22	37.3%	χ^2^ = 1.95	0.162
Odds ratio	1		1.630 (0.82 – 3.24)		β = 0.49	0.164

* Significant p values. Odds ratio reported as odds ratio (95% confidence interval).

The all-patient mean HADS-D subscale score was 7.54 ± 4.322. It is particularly important to note that the HADS-D score was notably higher in women (8.66 ± 3.717) than in men (6.87 ± 4.531, p = 0.004). About 54.2% of female and 47.5% of male patients exhibited a depression disorder, being mild in 30.5%, moderate in 16.9% and severe in 6.8% of females, while the respective percentages for males were mild in 24.8%, moderate in 16.8% and severe in 5.9% (Table 2). It should be noted that the distribution of the aforementioned depression symptomatology severity degrees did not statistically differ between genders (p = 0.841).

**Table 2:** Prevalence of anxiety and depression based on gender and severity

Severity of anxiety/depression	Hospital Anxiety and Depression Scale (HADS)													
	Anxiety subscale							Depression subscale						
	Total		Men		Women		p value	Total		Men		Women		p value
	n	%	n	%	n	%		n	%	n	%	n	%	
No disorder	82	51.3	61	60.4	21	35.6	0.002*	80	50	53	52.5	27	45.8	0.413
Mild disorder	39	24.4	18	17.8	21	35.6	0.012*	43	26.9	25	24.8	18	30.5	0.428
Moderate disorder	30	18.8	16	15.8	14	23.7	0.217	27	16.9	17	16.8	10	16.9	0.985
Severe disorder	9	5.6	6	5.9	3	5.1	0.821	10	6.3	6	5.9	4	6.8	0.832
Total	82	51.3	61	60.4	21	35.6	0.002*	80	50	53	52.5	27	45.8	0.413

* Significant p values (between men and women, χ^2^). HADS, Hospital Anxiety and Depression Scale.

The HADS-A subscale score analysis revealed that all-patient mean HADS-A subscale score was 7.59 ± 4.335 and women had a higher mean score of 8.2 ± 3.938, while the mean score in men was 7.18 ± 4.532 (p = 0.142). 64.4% of female and 39.6% of male respondents had anxiety symptoms (mild in 35.6%, moderate in 23.7% and severe in 5.1% of females, while the respective percentages for males were mild in 17.8%, moderate in 15.8% and severe in 5.9%) (Table 2). According to the anxiety severity degree data, the prevalence of anxiety was considerably higher in women (p = 0.014), with this difference being more significant in the mild anxiety group (p = 0.012). Logistic regression analysis demonstrated that women had an elevated risk of having an anxiety disorder, with an OR of 2.76 (Table 2).

There was no significant association between patient age and anxiety severity. However, a weak positive significant correlation was found between patient age and depression severity (p = 0.233, p = 0.003). In addition, it was determined that male patients who developed depression were on average younger than those without the disease, with a mean age of 58 years and 66 years, respectively (p = 0.005). The age with the highest risk of developing depression was determined to be between 55 and 62 years of age for males, whilst 95% of female patients who developed depression were between 66 and 75 years of age. A subsequent gender-based analysis showed that there was a significant weak positive correlation between male patient age and depression severity (p = 0.212, p = 0.033) and a weak negative correlation between male patient age and anxiety severity (p = - 0,278, p = 0.005). In contrast, the data analysis in women did not demonstrate any statistical association, thus meaning that depression and anxiety severity is similar for women of all age after MI. (Table 3)

**Table 3:** Cardiovascular risk factor characteristics distributed by gender

Risk factors	Men		Women		p value
	n = 101	%	n = 59	%	
Current smoker	21	20.8	4	6.8	0.019*
Physical inactivity	49	48.5	27	45.8	0.737
Diabetes mellitus	21	20.8	21	35.6	0.040*
Hypertension	87	86.1	57	96.6	0.033*
Hypercholesterolemia	52	51.5	39	66.1	0.072

* Significant p values (between men and women, χ^2^). MI, myocardial infarction.

Cardiovascular risk factor analysis showed an association between diabetes mellitus and HADS-D score in males who had a significantly higher median depression score compared to those who were not diabetic (10, IQR 5 - 11 vs. 5, IQR 3 - 9.75, p = 0.043). In contrast, female patients did not show any significant association between diabetes mellitus and emotional disorders. Hypercholesterolemia was associated with both higher median anxiety (8, IQR 6 - 12 vs. 6.5, IQR - 4 - 8, p = 0.02) and depression (9, IQR 7 - 12 vs. 7, IQR 4 - 8.75, p = 0.015) scores in women, while men did not show any association between the aforementioned factors (Table 4). Moreover, it was determined that arterial hypertension and body mass index were not, in any way, associated with anxiety or depression. The evaluation of patient smoking habits revealed that 15.6% of respondents were daily smokers (Table 3). Smoking was more prevalent amongst men than women (20.8% vs. 6.8%, p = 0.019). Furthermore, a higher HADS-A score was identified in male patients who did smoke (10, IQR 7.5 - 14) vs. 6.5, IQR 3 - 9, p = 0.002), whilst the HADS-A score did not differ between women who smoked or did not smoke (p = 0.311). Likewise, there was no statistically significant difference in the HADS-D subscale scores between smoking and non-smoking patients. Exercise habit analysis showed that the group of patients who did not exercise had a higher median HADS-D score than the group that exercised (9, IQR 6 - 12 vs. 5, IQR 3 - 9, p < 0.001). Gender-based analysis similarly revealed significantly higher median HADS-D scores in male (7.5, IQR 5 - 12 vs. 4, IQR 3 - 8, p = 0.002) and female patients (9, IQR 7.25 - 11.75 vs. 8, IQR 4 - 10, p = 0.027) who were hypodynamic. In contrast, the median HADS-A score did not significantly differ between females and males who exercised and did not exercise (p = 0.676) (Table 4).

**Table 4:** Cardiovascular risk factors and Hospital Anxiety and Depression Scale score

Risk factors		HADS-A		HADS-D	
		Median (interquartile range)		Median (interquartile range)	
		Men	Women	Men	Women
Current smoker	Yes	10 (7.5 – 14)	9 (7.25 – 13)	5 (2 – 11.5)	6 (4 – 11.75)
	No	6.5 (3 – 9)	8 (5 – 10)	6 (4 – 10)	8 (7 – 11)
	p value	0.002*	0.311	0.473	0.439
Physical inactivity	Yes	7.5 (4 – 11)	7 (5 – 10.75)	7.5 (5 – 12)	9 (7.25 – 11.75)
	No	7 (3 – 10)	8 (6 – 10)	4 (3 – 8)	8 (4 – 10)
	p value	0.286	0.364	0.002*	0.027*
Diabetes mellitus	Yes	7 (4 – 9)	8 (6 – 11)	10 (5 – 11)	9 (7 – 13)
	No	7 (3 – 11)	8 (5 – 10)	5 (3 – 9.75)	8 (5.75 – 10.25)
	p value	0.943	0.537	0.043*	0.283
Hypertension	Yes	7 (4 – 10)	8 (5.5 – 10.5)	6 (3 – 10)	8 (6.5 – 11)
	No	6 (2.75 – 10.75)	N/A	8 (4.75 – 10.25)	N/A
	p value	0.756	N/A	0.287	N/A
Hypercholesterolemia	Yes	7 (4 – 9)	8 (6 – 12)	6.5 (4 – 10)	9 (7 – 12)
	No	8 (3.5 – 12.5)	6.5 (4 – 8)	6 (3 – 10.5)	7 (4 – 8.75)
	p value	0.2	0.02*	0.859	0.015*

* Significant p values. HADS: Hospital Anxiety and Depression Scale; HADS-A: Hospital Anxiety and Depression Scale-Anxiety; HADS-D: Hospital Anxiety and Depression Scale-Depression.

## Discussion

This study assessed gender differences regarding the associations between emotional disorders and MI that had occurred less than one month before the initial assessment. Our investigation showed that 71.4% of female and 60.4% of male patients had some type of emotional mental health problem after having been diagnosed with MI. Subsequently, we observed an elevated risk of concomitant emotional disorders in women, in comparison to men (p = 0.006). Likewise, a gender-associated difference was displayed by Carvalho et al. who used the same HAD scale and found depression symptoms in 17.5% of adult inpatients with cardiovascular disease and anxiety symptoms in 32.5% and, amongst these, the highest prevalence of mental disorders were also associated with female gender (anxiety: p = 0.002; depression: p = 0.022).^13^ Although the incidence of depression in women in society is nearly double than that in men,^14^ it is of utmost importance to stress that this gender-based discrepancy in society is quite probably irrelevant to our study, as the mean age of women in our study was 70 years and the incidence of depression in women after menopause (when reproductive hormones stabilize) is similar to that in men.^15^ The high prevalence of emotional disorders that we observed may be partially explained by the fact that we only assessed those with a more severe condition, i.e., MI. A similar study that also used the HAD scale, but assessed dermatological patients in the same region (Vilnius city, Lithuania), found the prevalence of mental disorders to be higher than in other comparable studies, although lower than that observed in our study.^16^ Another noteworthy explanation for the high prevalence might be the fact that mental health problems in Lithuania are particularly widespread, as demonstrated by the suicide rates that are amongst the highest worldwide.^17^

It is particularly important to note that the HADS-D score was especially higher in women. Although we did not assess the impact of depression on patient outcomes, it is nonetheless necessary to stress the predictive influence of depressive symptoms in acute coronary syndrome [ACS]. A meta-analysis including 22 studies, carried out by Van Melle et al.,^18^ concluded that depression is associated with a two-fold increase in mortality following MI. Furthermore, depression is associated with worse long-term outcomes after MI. For example, it was determined that moderate or high stress at the time of the MI is associated with an increased two-year mortality and an increased risk of angina in the first year.^19^ Bush et al.^20^ prospectively studied patients with MI who survived to discharge and determined that the highest mortality rates were observed in patients with the most severe depressive symptoms. Moreover, the ENRICHD study^21^ also concluded that depression increases the risk of all-cause mortality for 30 months, even after adjusting for confounders. After the extensive review of 53 studies and four meta-analyses, the American Heart Association [AHA] stated that depression is an individual risk factor for adverse medical outcomes in patients with acute coronary syndrome.^21^ Depression is an important risk factor that should be taken into consideration, not only after ACS but prior to CAD as well. Results of an 11-cohort study meta-analysis by Rugulies et al.^11^ support this statement, since they concluded that clinical depression was a strong predictor of the development of coronary heart disease in an initially healthy population. Furthermore, another study demonstrated that depression was a stronger CHD predictor, especially for women (p = 0.002).^22^

Our study revealed that women had a markedly elevated risk of having anxiety disorder. It should be highlighted that the prognostic significance of anxiety raises discussions, since some studies suggest that post-MI anxiety symptoms were not an independent prognostic risk factor for new cardiovascular events or death.^23^ Moreover, according to Hosseini et al.,^24^ post-MI anxiety does not predict long-term quality of life in MI survivors. Nonetheless, we believe that post-MI anxiety should be taken into consideration in clinical practice, since it has been shown that not only depression but also pre-myocardial anxiety in the preceding 2 hours increase 10-year mortality rates in those aged > 65 years.^25^ Moreover, Paine et al.^26^ recently published an article stating that women with anxiety and no CAD history had higher rates of ischemia than women without anxiety. Since women are more prone to anxiety, it is important to mention that many CAD symptoms (for example, fatigue, chest pain and shortness of breath) overlap with anxiety symptoms and might mask CAD. This is more evident in women than men and contributes to the referral to other specialists and, thus, diagnostic delays.^27^

Although a recent publication by Feng et al.^1^ determined that especially those women between 45 and 64 years of age had the greatest risk for anxiety when it comes to cardiovascular disease, our findings did not support this conclusion. First, our study showed that women had the highest probability to develop anxiety from 68 to 75 years of age. Second, the analysis showed that age did not have any influence on either anxiety or depression prevalence in women. On the other hand, there was a significant association between age in men and depressive symptomatology prevalence and it was shown that a relatively younger population, aged 55 to 62 years, had the highest risk of developing depression. Furthermore, male patients showed a significant weak positive correlation between age and depression severity and a weak negative correlation between age and anxiety severity.

The cardiovascular risk factor analysis showed that a higher anxiety score was identified in male patients who smoked, whereas the HADS-A score did not differ between women who smoked and did not smoke. Similarly, a significantly higher HADS-D score was found in those patients who were hypodynamic. Also, an association between diabetes mellitus and the HADS-D score was evident and men who had diabetes mellitus also had a significantly higher depression score, whereas female patients did not show any significant association between diabetes mellitus and emotional disorders. Although our analysis did not demonstrate any association between hypertension and mental disorders, another study listed depression as being associated with several known prognostic factors, such as a history of treatment of hypertension, diabetes, advanced Killip Class and left ventricular ejection fraction of 35% or less.^28^ We would also like to address the association found between elevated anxiety and depression levels and hypercholesterolemia in females. A quite recent experimental study by Engel et al.^29^ aimed to investigate this pathophysiological association and concluded that depressive-like behavior in hypercholesterolemic mice is accompanied by alterations in the monoaminergic metabolism, providing new evidence about the association between hypercholesterolemia and depression.

It is of paramount importance to mention the need for routine screening for depression since it is also associated with decreased adherence to medications^30^ and a three-fold increase in the risk of noncompliance with medical treatment regimens.^31^ Moreover, it leads to significantly reduced quality of life^32^^,^^33^ and higher healthcare costs.^34^ All patients should be screened within one month of MI. The AHA recommends using Patient Health Questionnaire-2, which consists of one question seeking to identify a depressive mood in the preceding two weeks and another for anhedonia in the preceding two weeks.^35^ If the answer is positive to either question, then the patient should be referred for a more thorough clinical evaluation by a professional qualified in the diagnosis and management of depression or screened with the Patient Health Questionnaire-9, which has shown to be diagnostically superior in patients with CHD.^36^ In contrast, there are no specific guidelines from the AHA for anxiety disorder screening in CHD. This can be partially due to the high prevalence of anxiety symptoms in angina and MI. Furthermore, it has been shown that anxiety rating scales have relatively high false positive scores that result in reduced cost-effectiveness of routine screening.^37^

Both depression and anxiety treatment options include cardiac rehabilitation and exercise therapy, disease management programs, cognitive behavior therapy and pharmacotherapy.^38^ Data from the Secondary Prevention in Uppsala Primary Health Care study further support the heart-helping benefits of cognitive-behavioral therapy since at follow-up, the psychotherapy intervention group had 45% fewer recurrent heart attacks and a 41% lower rate of both non-fatal and fatal first recurrent cardiovascular events than the group receiving traditional care.^39^ On the other hand, there are still ongoing discussions concerning the optimal treatment algorithm, as a few studies have had disappointing results concerning behavior therapy. For example, the ENRICHD study found that a six-month intervention focused on treating patients' depression made patients feel better, but had no positive impact when it came to preventing repeat heart attacks or death.^40^

Possible limitations of our study include unequal sample sizes between genders, with the male group being larger. However, this gender inequality reflects the real rates of patients with MI admitted to hospitals in Lithuania. Second, the absence of a control group can be considered a limitation, though we have attempted to mitigate this by discussing and comparing our data with results of previous similar studies. Third, the study design did not include mental health evaluation by a psychiatrist. Finally, our study was not a longitudinal one and patients were not reassessed several times to determine a more long-term association between MI and mental disease.

## Conclusions

MI is especially closely associated with anxiety and depression. More than two-thirds of patients with MI had a depression and/or anxiety comorbidity within the first month of MI. Women showed an elevated risk of having anxiety and/or depression disorder compared to men. Furthermore, both anxiety and depression severity had a tendency to be higher in women. In addition, depression severity increased with age in men, while anxiety severity decreased. In contrast, depression and anxiety severity are similar for women of all ages after MI. A higher depression score was associated with diabetes and physical inactivity, whereas a higher anxiety score was associated with smoking in men. Hypercholesterolemia was associated with both higher anxiety and depression scores, whereas a higher depression score was associated with physical inactivity in women.
